# Evaluation of Cell Cycle Arrest in Estrogen Responsive MCF-7 Breast Cancer Cells: Pitfalls of the MTS Assay

**DOI:** 10.1371/journal.pone.0020623

**Published:** 2011-06-03

**Authors:** Eileen M. McGowan, Nikki Alling, Elise A. Jackson, Daniel Yagoub, Nikolas K. Haass, John D. Allen, Rosetta Martinello-Wilks

**Affiliations:** 1 Translational Cancer Research Group, School of Medical and Molecular Biosciences, Faculty of Science, University of Technology Sydney, Sydney, New South Wales, Australia; 2 Sydney Medical School, The University of Sydney, Sydney, New South Wales, Australia; 3 Flow Cytometry Unit, Garvan Institute of Medical Research, Sydney, New South Wales, Australia; 4 Centenary Institute, Sydney, New South Wales, Australia; Beth Israel Deaconess Medical Center, United States of America

## Abstract

Endocrine resistance is a major problem with anti-estrogen treatments and how to overcome resistance is a major concern in the clinic. Reliable measurement of cell viability, proliferation, growth inhibition and death is important in screening for drug treatment efficacy *in vitro*. This report describes and compares commonly used proliferation assays for induced estrogen-responsive MCF-7 breast cancer cell cycle arrest including: determination of cell number by direct counting of viable cells; or fluorescence SYBR®Green (SYBR) DNA labeling; determination of mitochondrial metabolic activity by 3-(4,5-dimethylthiazol-2-yl)-5-(3-carboxymethoxyphenyl)-2-(4-sulfophenyl)-2H-tetrazolium (MTS) assay; assessment of newly synthesized DNA using 5-ethynyl-2′-deoxyuridine (EdU) nucleoside analog binding and Alexa Fluor® azide visualization by fluorescence microscopy; cell-cycle phase measurement by flow cytometry. Treatment of MCF-7 cells with ICI 182780 (Faslodex), FTY720, serum deprivation or induction of the tumor suppressor p14ARF showed inhibition of cell proliferation determined by the Trypan Blue exclusion assay and SYBR DNA labeling assay. In contrast, the effects of treatment with ICI 182780 or p14ARF-induction were not confirmed using the MTS assay. Cell cycle inhibition by ICI 182780 and p14ARF-induction was further confirmed by flow cytometric analysis and EdU-DNA incorporation. To explore this discrepancy further, we showed that ICI 182780 and p14ARF-induction increased MCF-7 cell mitochondrial activity by MTS assay in individual cells compared to control cells thereby providing a misleading proliferation readout. Interrogation of p14ARF-induction on MCF-7 metabolic activity using TMRE assays and high content image analysis showed that increased mitochondrial activity was concomitant with increased mitochondrial biomass with no loss of mitochondrial membrane potential, or cell death. We conclude that, whilst p14ARF and ICI 182780 stop cell cycle progression, the cells are still viable and potential treatments utilizing these pathways may contribute to drug resistant cells. These experiments demonstrate how the combined measurement of metabolic activity and DNA labeling provides a more reliable interpretation of cancer cell response to treatment regimens.

## Introduction

Drug resistance is a major problem in cancer research. As a result there is an increasing emphasis on understanding the mechanism of drug metabolism in the control of cancer cell proliferation, viability and death to enable improved drug therapies for patients. A major interest in our laboratory is to understand anti-estrogen treatment resistance in breast cancer. We have been using three methods to inhibit estrogen action: anti-estrogen ICI 182780 (Faslodex); inhibition of sphingosine kinase activation of the estrogen receptor using the immunosuppressive drug FTY720; re-activation of the tumor suppressor p14ARF-p53 pathway. Faslodex is currently in clinical use as an anti-hormone therapy and also commonly used in the laboratory to study estrogen action [1]. This compound binds and down-regulates the estrogen receptor (ER) and induces quiescence by stopping cell cycle progression from G0/G1 into S-phase [2]. The drug FTY720 is a sphingosine analogue and is currently in clinical use as an immunosuppressant for multiple sclerosis [3]. FTY720 acts by inhibiting sphingosine kinase (SK) activity [4]. SK1 is causally associated with endocrine resistance, in part, by enhancing estrogenic proliferative activity [5]. FTY720 has been shown to directly affect mitochondria and trigger apoptosis [6]. Mimetics of p14ARF, such as nutlin 3, which activate the p53 pathway, are now in clinical trial for cancer therapy [7]. It is well documented that p14ARF binds to hdm2 (homolog of mouse mdm2) and stabilizes p53 protein thus inhibiting cell cycle progression [8]. Importantly for our research, p14ARF is associated with anti-estrogen action whereby both mdm2 and p53 bind to the estrogen receptor (ER) and inhibit estrogen activity in breast cancer cells [9], [10], [11]. In our hands p14ARF inhibits cell proliferation and induces hypertrophy in MCF-7 cells.

Assays commonly used by cancer researchers to assess cell number, cell viability and cytotoxicity in response to drug treatment include the metabolic MTT [12] or MTS [13] assays that measure mitochondrial enzymatic activity that occurs only in viable, proliferating cells. However, cell cycle arrest does not necessarily result in metabolic dysfunction. In some instances a perturbation of cellular processes resulting in increased metabolic activity can provide a false positive readout of cell response to treatment [14]. Alkylators and anthracyclines, for example, damage proteins as well as DNA, resulting in energy-dependent protein degradation and resynthesis. An alternative approach to determining cell cycle arrest utilizes DNA labeling with SYBR®Green I (SYBR) or other dyes such as CyQuant, whereby DNA content can be accurately measured by fluorescence spectrometry [15], [16], [17], [18]. This assay more accurately measures cell number, proliferation and cell cycle arrest as analyzed by DNA content. The EdU click-IT assay is designed to measure only proliferating cells in live cell culture. Newly synthesized DNA incorporates 5-ethynyl-2′-deoxyuridine (EdU) nucleoside analog, similar to the BrdU assay, but less toxic.

In this study we examine the reliability of MTS, SYBR-DNA labeling and EdU *in vitro* assays to determine cell cycle arrest in estrogen sensitive MCF-7 cells following treatment with ICI 182780, FTY720 or following the induction of p14ARF. We discuss the practical advantages/disadvantages of using mitochondrial activity assays and DNA labeling assays in parallel as fast, effective methods of screening anti-proliferative effects following drug intervention to determine cell fate. Furthermore, understanding the mechanism of drug action at the cellular or nuclear level will aid in the design of combinatorial drug therapies for patients with unmet needs.

## Results

### Comparative analysis of Cell counts, MTS and SYBR-DNA labeling assays

**Figure 1:**
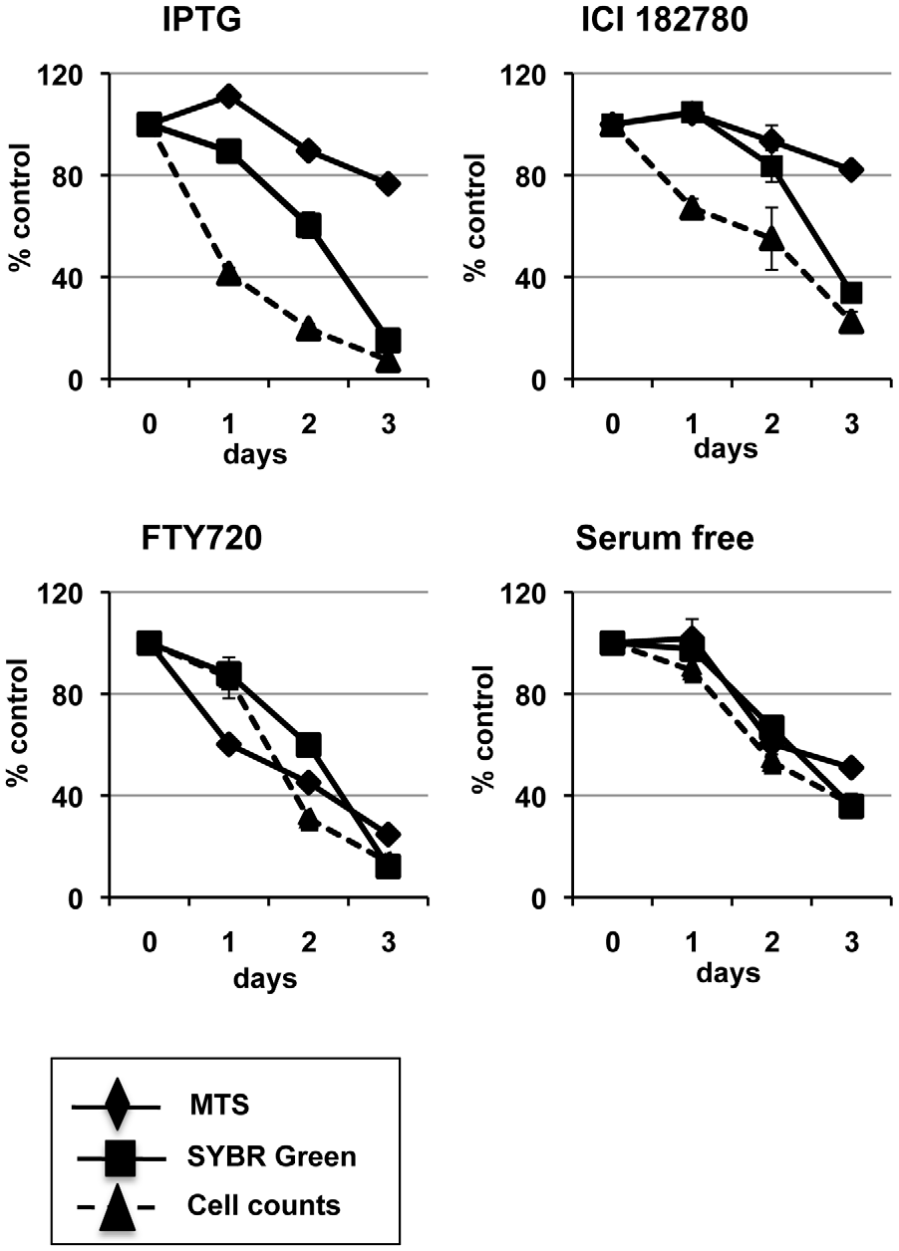
Comparative analysis of MCF-7 cell viability, cell number and mitochondrial activity. Cells were treated with 5 mM IPTG, 10 nM ICI 182780, 5 µM FTY720, or serum deprived (serum free) 48 h post-seeding. Using the Trypan Blue exclusion method cells were harvested and viable cells counted using a haemocytometer at days indicated. MTS and SYBR-DNA assays were performed, as detailed in materials and methods, at days indicated. The treatment results are shown as a percentage of the uninduced vehicle control (±SE) correlating with viable cell number (Trypan blue counts), colorimetric measurement (MTS), and fluorescent intensity (SYBR assay). Each experiment was performed in triplicate at least 3 times with similar results.

In this study we analyzed the anti-proliferative effects of the pure anti-estrogen ICI 182780, p14ARF-p53 induction, FTY720 drug treatment and serum starvation in MCF-7 cells. These experiments were conducted with MCF-7 cells showing p14ARF induction with IPTG. Without IPTG induction, these MCF-7 cells retained all the characteristics of native breast cancer cells including their responsiveness to anti-estrogen treatment (unpublished data). We compared viability of cells post-treatment using the Trypan Blue exclusion assay measured by direct cell counts, the MTS assay by colorimetric measurement, and SYBR-DNA assay measured by cell fluorescence. For simplicity we expressed the results as a mean percentage of control ± SE to allow for direct comparison of all assays (Fig. 1).

#### Induction of p14ARF with IPTG

As shown in Figure 1 (IPTG), induction of p14ARF with IPTG dramatically inhibited cell proliferation by 60–80% (cell counts) within the first 2 days compared to control cells when a 20–50% reduction was observed by SYBR green fluorescent assay. Interestingly, the MTS assay showed an increase in mitochondrial activity at 24 h (115%) and the activity was only suppressed by 10% at day 2 despite a significant reduction in cell number. The SYBR assay showed an 85% inhibition in proliferation by day 3, yet the MTS assay showed only 20% suppression in metabolic activity. The trypan blue count confirmed that viable cell numbers did not increase in the IPTG condition when compared to the controls (Fig. 1, IPTG).

#### *ICI 182780 treatment*

Upon treatment with the anti-estrogen ICI 182780 the SYBR green assay showed a deficit of 20% and MTS assay showed little change within the first 2 days despite a 30–50% reduction in cell number (Fig. 1, ICI 182780). Similar to IPTG results, when cells were treated with ICI 182780 the SYBR assay showed a marked inhibition of proliferation (60%) by day 3, whereby the MTS assay only showed 20% suppression in mitochondrial activity. The trypan blue count confirmed the number of viable cells did not increase compared to control (Fig. 1, ICI 182780).

#### FTY720 treatment

When cells were treated with FTY720, all three assays, Trypan blue counts, SYBR and MTS assays showed 70–80% suppression in proliferation by day 3 (Fig. 1, FTY720). However, in contrast, the FTY720 treatment significantly reduced mitochondrial activity (MTS) on day 1 (40% of control) with little impact on SYBR DNA content (Fig. 1, FTY720). This result is consistent with previous reports showing FTY720 directly slows mitochondrial activity [6].

#### Serum free treatment

When cells were deprived of serum there was no difference in results yielded from the Trypan Blue, MTS or SYBR assays (Fig. 1, Serum free).

Comparatively, when assessing the different treatment regimes, our results showed that the MTS and SYBR assays might not be as reliable a measure of cell proliferation as cell counts.

### Flow cytometric analysis of ICI 182780 or p14ARF anti-proliferative effects

**Figure 2:**
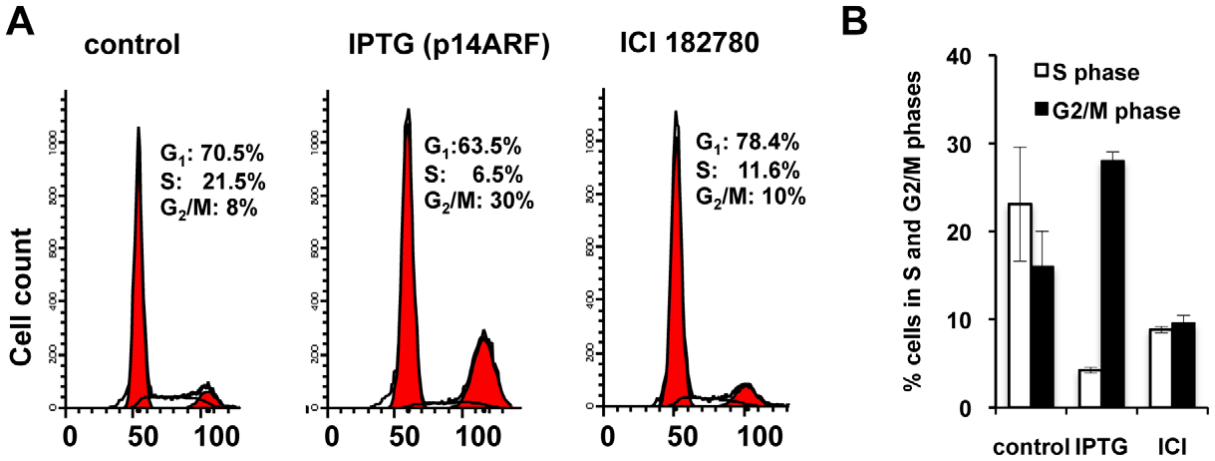
Flow cytometric analysis of cell cycle phases post ICI 182780 and IPTG treatment. Cells were treated with 10 nM ICI 182780 or 5 mM IPTG (p14ARF-induction) 48 h post seeding. At 48 h post-treatment cells were harvested, stained with propidium iodide solution as described in materials and methods and analysed for cell cycle distribution by flow cytometry using Modfit software. A. Fluorescence histograms showing cell cycle distribution of control, IPTG and ICI 182780 treated MCF-7 cells (representative experiment). B. Representative column graph showing the percentage of cells (± SD) in each cell cycle. This experiment was performed three times with three different cell lines showed similar results.

The above observations showed distinct discrepancies between the MTS and SYBR-DNA assay readouts and cell counts when cells were treated with ICI 182780 and IPTG (induction of p14ARF) at days 1 and 2. Flow cytometric analysis confirmed cell cycle inhibition post treatment with ICI 182780 or IPTG (Fig. 2), concordant with the cell count results (Fig. 1). Flow cytometric results presented additional information on cell behaviour post drug treatment; IPTG induction of p14ARF significantly reduced the S-phase peak with an accumulation of cells in the G1 and G2/M cell-cycle phases whereas ICI 182780-treated cells accumulated in the G1/GO phase (Fig. 2A). These experiments were repeated with three different p14ARF-clonal cell lines with similar results to ensure that G2/M accumulation post-IPTG treatment was not due to aberrant clonal variation (Fig. 2B). Our results are consistent with the published literature, which demonstrate ICI 182780 induces quiescence (G1/G0) in MCF-7cells [2], and p14ARF-p53 arrests cells in both G1 and G2/M cell-cycle phases [19].

### EdU-DNA incorporation post-treatment with ICI 182780 and IPTG (p14ARF)

**Figure 3:**
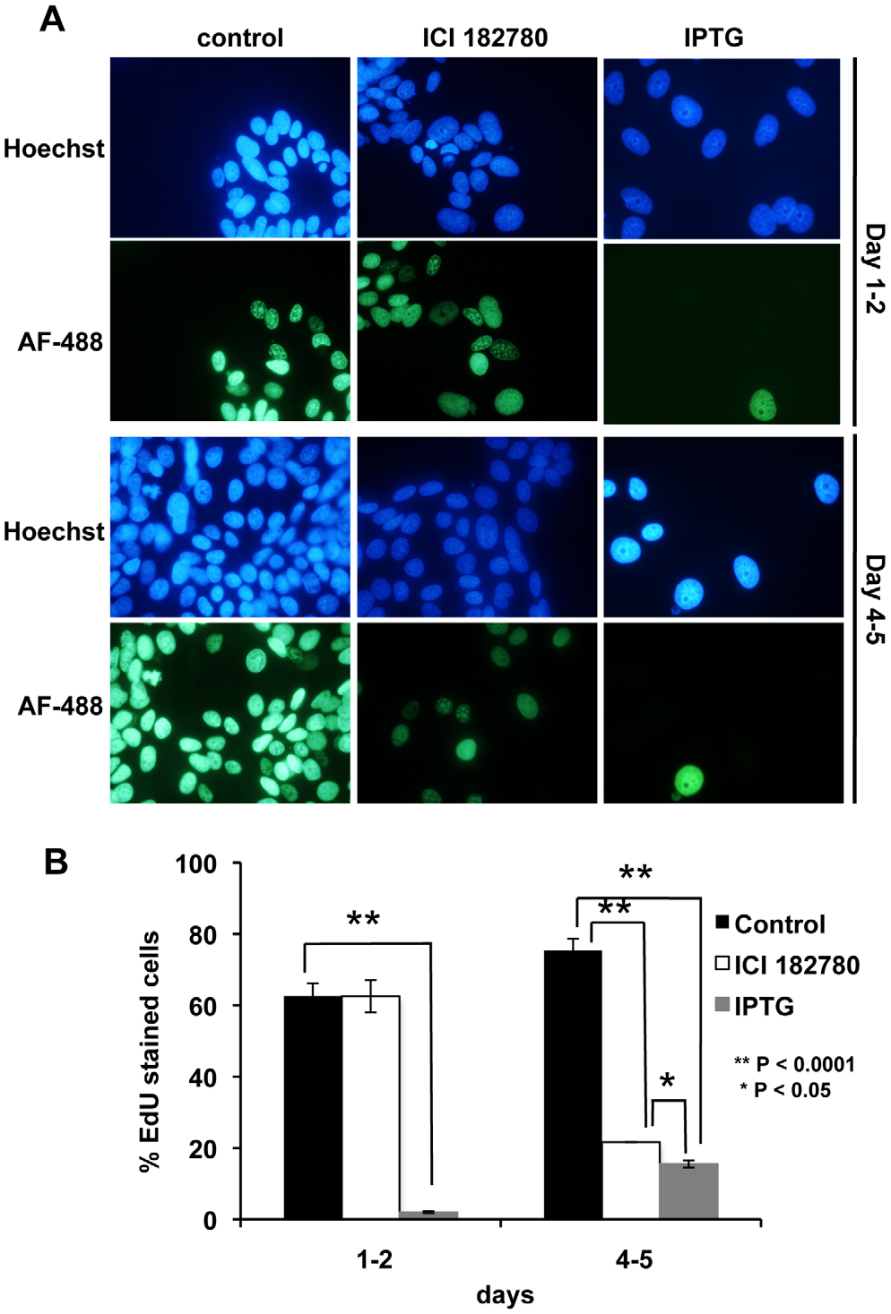
EdU incorporation post ICI 182780 and IPTG (p14ARF) treatment. Cells were seeded on cover slips and treated with 5 mM IPTG or 10 nM ICI 182780 24 h post-seeding. EdU was added to the medium on days 1 and 4 and cells were incubated for a further 20 h. EdU incorporation was visualized by staining with Alexafluor 488 (green). The nucleus was stained with Hoechst 33342 (blue) and images were taken on a Nikon fluorescence microscope (magnification ×200). Column graph shows % cells staining for EdU compared to Hoechst 33342 stained nuclei (± SE). Experiments were performed in triplicate (duplicate biological experiments). A minimum of 500 cells was counted for each treatment.

To measure DNA synthesis, in particular the percentage of cells in S-phase, EdU was added directly to the MCF-7p14ARF cell culture medium 24 h and 96 h post-treatment with IPTG or ICI 182780 and cells were incubated for a further 20 h. This allowed the visualization of individual cell proliferation captured over a 20-hour window. At 24 h post-IPTG treatment, 5% of IPTG-treated cells synthesized DNA (Fig. 3), suggesting rapid repression of DNA synthesis upon reactivation of the p14ARF pathway in MCF-7 cells, which is consistent with the cell count data, but not with MTS or SYBR green assays (Fig. 1). Reduced proliferation was maintained for 96 h–116 h (Fig. 3). These later-day results were in agreement with the cell counts and SYBR-DNA assay as shown in Figure 1, but not with the MTS assay. ICI 182780-treated cells showed a significant inhibition in DNA synthesis by day 4–5 post-treatment when only 20% of these cells incorporated EdU when compared to control cells (P<0.0001; Fig. 3). Interestingly, ICI 182780 treatment showed a delayed cell cycle arrest mechanism when compared to p14ARF induction with IPTG, which showed an early inhibition of DNA synthesis by day 1–2 (P<0.0001; Fig. 3). This is consistent with the Flow analysis data and previously reported literature where ICI 182780 effective blocks the cell cycle only in G1/G0 phase [2].

### P14ARF and ICI 182780 increase mitochondrial activity in MCF-7 cells

**Figure 4:**
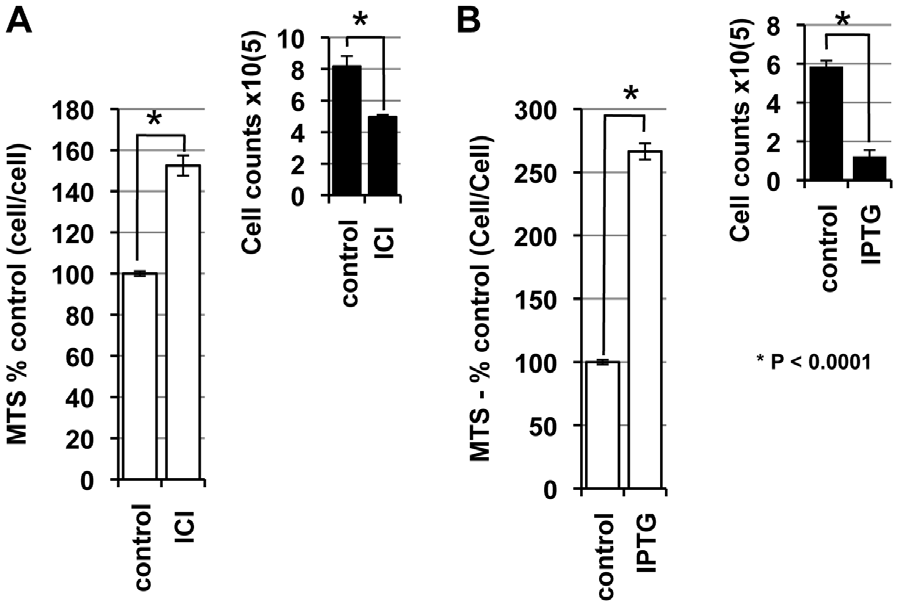
ICI 182780 and p14ARF increase mitochondria activity. Cells were treated with 10 nM ICI 182780 (**A**) or 5 mM IPTG (**B**), 48 h post seeding. At 72 h post treatment cells were counted (see inset) and equal number of cells plated in 96 well plates. Mitochondrial activity was measured using the MTS assay. Treatment results were presented as percentage of control (± SE) in column graphs. Each experiment was performed in duplicate at least 3 times.

We have shown significant differences between the MTS and SYBR assay readouts when investigating the anti-proliferative effect of ICI 182780 and p14ARF-induction. We therefore determined mitochondrial activity on a cell-cell basis using the MTS assay. On day 3 post-treatment with ICI 182780, IPTG or control, cells were harvested and equal numbers of treated and untreated cells were seeded into 96 well plates and mitochondrial activity determined by MTS assay (Fig. 4). On a comparative cell-cell basis IPTG-treated cells showed a significant (2.6 fold) increase in mitochondrial activity compared to untreated cells (P<0.0001; Fig. 4B). ICI 182780 also resulted in a significantly increased mitochondrial activity (1.6 fold) per cell (P<0.0001; Fig. 4A).

### Induction of p14ARF increases mitochondrial biomass with no loss of mitochondrial membrane potential

**Figure 5:**
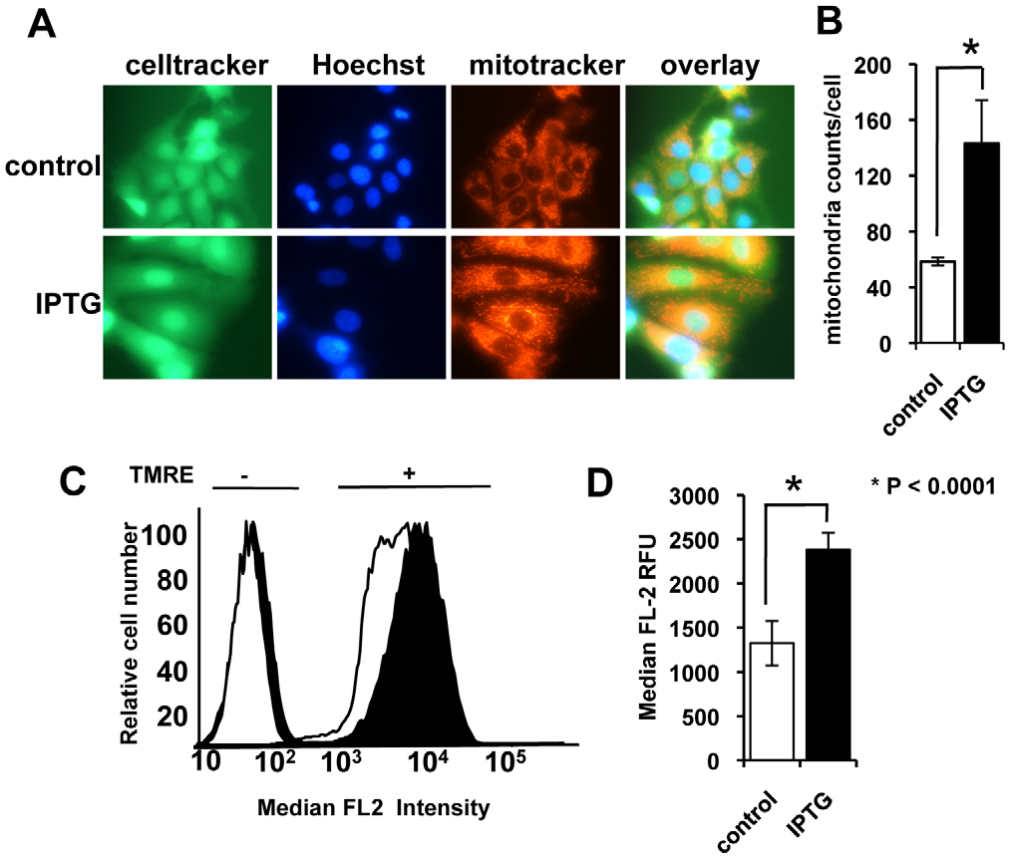
Induction of p14ARF increases mitochondrial biomass and maintains membrane potential. **A.** Cells were treated with 5 mM IPTG 48 h post-seeding. At day 3 post-IPTG treatment, live cells were incubated with Mitotracker (red), CellTracker (green) and Hoechst 33342 (blue) and imaged using an inverted fluorescent microscope (magnification ×400). Cells treated with IPTG noticeably increased in size. **B.** Images processed by high content imaging (magnification ×200) and mitochondria (per cell) counted using BD Attovision™ software. **C.** Cells were treated with 5 mM IPTG 48 h post-seeding. On day 3 post-IPTG-treatment cells were stained with TMRE for 15 min (+), or left unstained (−) and run through a flow cytometer (IPTG = black, control = white). TMRE-IPTG-treated cells showed increased fluorescence intensity compared to the TMRE-control cells, which is indicative of an increase in ΔΨ_mt_ in IPTG-treated cells. **D.** The median FL2 relative fluorescence units (RFU) of control and IPTG treated cells (day 3) were determined by flow cytometry. The column graph shows the median RFU of TMRE-stained cells minus unstained cells (±SE). This experiment was performed at least twice in triplicate.

To understand the mechanism underlying the increased metabolic activity in MCF-7 cells with p14ARF induction, mitochondrial content and the mitochondrial membrane potential in treated and untreated cells were analysed. At day 3 post-IPTG-treatment, MitoTracker™ red, CellTracker™ green, and Hoechst 33342 was added to the cell cultures and live cells were imaged (Fig. 5A). A noticeable increase in cell size by day 3 was observed concomitant with an increase in mitochondria density per cell; this increase in cell size is consistent with the senescence-phenotype. Mitochondria cell number was quantified using the BD pathway Bioimager and Attovision software (Fig. 5B). A 3-fold increase (P<0.001) in mitochondrial counts post-p14ARF induction compared to control cells was observed.

#### Measurement of mitochondrial membrane potentiality (ΔΨ_mt_)

Tetramethylrhodamine ethyl ester (TMRE) is a red-orange fluorescent dye, which is readily taken up by active mitochondria. The amount of orange/red fluorescence is indicative of ΔΨ_mt_. To determine whether the mitochondria in our studies were functional we used TMRE uptake as a measure of ΔΨ_mt_. IPTG treated cells showing an increased TMRE incorporation at day 3 compared to untreated (TMRE-control) cells (P<0.001), confirmed that ΔΨ_mt_ was significantly increased in p14ARF expressing cells (Fig. 5 C and D), consistent with an increased biomass of functional mitochondria. These results strongly support the conclusion that the increase in metabolic activity, as demonstrated by MTS assays, was due to an increase in the numbers of functional mitochondria.

## Discussion

Large scale screening of drug effects on cell fate in cell culture is an important initial step to determine their potential efficacy in clinical application. Two screening assays commonly used to assess cell proliferation are the MTS assay, which measures dehydrogenase enzyme activity found in metabolically active cells, and SYBR-DNA labelling to determine DNA content. This study highlights the pitfalls researchers encounter by relying on one or both methods to determine cell viability or proliferation. Although we showed that SYBR-DNA is generally a more reliable, reproducible method to assess the anti-proliferative activity of drugs compared to the MTS assay over time, there are distinct discrepancies at early time points (days 1–2) between SYBR-green assay readouts and cell counts. In contrast, we clearly showed that the MTS assay is not always a reliable method to detect cell proliferation and is dependent on the drug of interest mode of action.

Nevertheless, each method can provide valuable information on drug efficacy. Here we show that initial screening using both methodologies offers some insight in cell behavior and presents a more informative picture of cell fate post drug treatment. Specifically, in this report we showed that the MTS assay is not on its own trustworthy as a method to measure the anti-proliferative activity of ICI 182780 which is clinically used for the treatment of advanced breast cancer [20], nor is it a reliable method to assess the anti-proliferative capacity of p14ARF-p53, an important tumor suppressor pathway. Although both treatments inhibit cell proliferation, we showed that the cells do not die and, indeed, showed increased metabolic activity.

Using p14ARF-induction as an example, SYBR-DNA labeling correlates favorably over time with viable cell number estimation by the Trypan Blue exclusion viable cell assay. Parallel studies using the MTS assay again indicated that although cells expressing p14ARF cells stopped proliferating, the viability of the cells was not strongly impacted. In fact, as with ICI 182780 treatment there was a clear increase in the metabolic activity of these cells. Information gained from Flow cytometric analyses of the cell-cycle-parameters and EDU live cell labeling provides additional insight on cell behaviour dependent on drug treatments. Inhibition of cell proliferation by p14ARF and ICI 182780 clearly showed different modes of action of cell-cycle phase repression, potentially contributing to latent recurrence or irreversible inhibition of cell proliferation post-drug treatment.

One of the major problems with current breast cancer treatments is the high rate of resistance to current therapy. For example, hormone therapies such as ICI 182780 used to treat breast cancer could also be called cytostatic therapy. Drug treatments such as ICI 182780 potentially contribute to latent resistant breast cancer, as we have shown cells retain viability and are metabolically active post-treatment. Further support for this idea is provided by our previous results showing that estrogen and progestins can reverse the anti-estrogenic effects of ICI 182780 in culture [21]. Drugs that target alternative signaling pathways, such as FTY720, may prove to be more effective alone, or in combinational therapies, in obtaining controlled cell death and tumor regression. Initial high throughput screening combining metabolic activity assays with DNA-labeling and Flow cytometric analyses would provide more reliable preliminary interpretation of cell reactions to drug regimens and also benefit the identification and design of novel therapeutic drug combination therapies.

## Materials and Methods

### Cell lines and culture conditions

MCF-7 epithelial breast cancer cells (ATCC 30–4500K) were maintained in complete medium: DMEM supplemented with 10% fetal bovine serum and penicillin/streptomycin. MCF-7 cells were stably transfected with p14ARF (MCF-7p14ARF) using the IPTG inducible system (Stratagene) as previously described [22]. Cells were maintained in hygomycin B (200 µg/mL and geneticin (200 µg/mL). Expression of p14ARF was induced by adding 5 mM IPTG to the cell medium and incubated at 37°C. Addition of 10 nM ICI 182780 (Faslodex) (Santa Cruz Biotechnology, Inc) and 5 µM FTY720 (Cayman Chemicals, Michigan, USA) were used to induce cell cycle arrest. All cells were tested mycoplasma free using the mycoAlert Kit (Lonza, Australia).

### Cell proliferation assays

#### MTS assay 1

Cells were seeded in 96-well plates at a density of 500 cells/well (dependent on the cell line) in 50 µL medium and left to attach for 48 h at 37°C. Subsequently, cells were exposed to drugs in an additional 50 µL medium. The first lane and column contained medium only to negate edge effects due to evaporation of the medium, the second lane was untreated controls (no drug). Cultures were further incubated at 37°C after which cells were assayed with MTS at time intervals 1–3 days as indicated. Measurements were made in accordance with the manufacturers instructions (Promega Corp., Madison, WI). In brief, 100 µL MTS reagent was added directly to the wells and cells incubated at 37°C for a minimum of 2 h. Assessment of metabolic activity was recorded as relative colorimetric changes measured at 492 nm. The data was transferred to Microsoft Excel and analyzed. Background absorbance was corrected using triplicate sets of wells containing medium only (no cells) and MTS reagent as per experimental well. The results represent the mean ± SE of quadruple samples, expressed as a percentage of control. Each experiment was performed at least three times.

#### MTS assay 2

Cells were seeded in 6-well plates at a density of 10^5^ cells/well and treated with drug concentrations as indicated or 5 mM IPTG (to induce p14ARF expression). At time intervals, as indicated, cells were trypsinized, counted using a haemocytometer and an equal number of cells were added to a 96-well plate. MTS reagent was added to the cells and incubated at 37°C for a minimum of 2 h and analyzed as described above.

#### SYBR-DNA-labeling assay

The SYBR-DNA-labeling experiment was performed as described for the MTS assay 1. Assays were stopped at time intervals 1–3 days post treatment as indicated, by flicking off the medium and freezing the plate. Relative cell numbers were determined in a similar manner to the CyQuant assay (Invitrogen); cells were stained with 200 µL/well of SYBR® Green I (Invitrogen) 1∶4000 in a hypotonic lysis buffer (10 mM Tris HCl pH 8, 5 mM EDTA, 0.1% Triton X-100) for 4–7 days in the dark at 4°C and then quantified by flourimetry at 535 nm with 485 nm excitation, measured using a Wallac VICTOR3V plate reader (Perkin Elmer P/L Melbourne Vic, Australia). Treated cell fluorescence intensity was presented as a percentage of intensity of control cells.

#### Trypan Blue exclusion viable cell assay

Cells were trypsinized and resuspended in equal volumes of medium and Trypan blue (0.05% solution) and counted using a haemocytometer. Trypan blue dye (Invitrogen) exclusion was used to assess cell viability.

#### Click iT™ EdU Imaging Kit

The EdU (5-ethynyl-2′ -deoxyuridine) is a nucleoside analog of thymidine that is incorporated into DNA only during DNA synthesis allowing the visualization of newly synthesized DNA [23]. This imaging technology is a less toxic alternative to the BrdU incorporation assay. To perform the assay cells were plated on cover slips in 6-well plates and incubated at 37°C for 24 h before treatment with 5 mM IPTG, 10 nM ICI 182780, or 5 µM FTY720. At time intervals indicated cells were treated with 2.5 µM EdU, directly added to the culture medium, and incubated for a further 20 h at 37°C to ensure capture of the majority of proliferating cells. Following EdU addition for 20 h, cells were fixed with 4% paraformaldehyde and permeabilised using acetone for 5 min at −20°C. Incorporation of EdU was observed by incubating fixed cells with 2% BSA in PBS for 30 m and Alexa fluor 488 for a further 30 min under Cu(I)-catalyzed click reaction conditions, as described by the manufacturer. Cells were washed with PBS, and counterstained with Hoechst 33342 (1∶1000) in PBS before mounting on slides using Fluor mount (Sigma) for fluorescent microscopy.

#### Cell kinetic studies

Cells were seeded at 10^5^ per well in 6 well plates and treated with 10 nM ICI 182780, 5 mM IPTG or vehicle, 24 h post-seeding. Cell cycle distribution was assessed using flow cytometry as described previously [21]. Briefly, at 48 h post-treatment cells were harvested, washed with PBS, fixed in cold ethanol and stained with propidium iodide solution containing RnaseA. A. Flow cytometric analysis was performed on a FACSCalibur (Becton Dickinson) using Modfit LT analysis software (Verity Software House, Inc., Topsham, ME). A minimum of 20,000 cells was counted for each sample. Analysis was done with ModFit software.

#### Mitochondria detection

Cells were treated with IPTG or vehicle over a period of 3 days. Live cell cultures were incubated with MitoTracker® 50 nM (Invitrogen) as per manufacturer instructions Cells were imaged using phase contrast and fluorescence microscopy using an Eclipse Ti-U inverted fluorescent microscope (Nikon, Tokyo, Japan) with a 5 mega-pixel cooled camera (×400 magnification). Mitochondrial counts, size, and distribution were measured using Pathway Bioimager™ analyzed with Attovision software (×200 magnification).

#### Mitochondrial membrane potential

The ΔΨ_mt_ was measured by flow cytometry using with 488 nm excitation and 670 nm emission. Log scale fluorescence histograms were analyzed for median relative fluorescent unit (RFU) intensity using Flow Jo software.

#### Statistical analyses

All data were analysed by the Students t-test using Microsoft Excel, unless otherwise stated.
